# SEX HORMONES ARE ASSOCIATED WITH BONE DENSITY AND PHYSICAL FUNCTION IN WOMEN RECOVERING FROM HIP FRACTURE

**DOI:** 10.1093/geroni/igad104.1442

**Published:** 2023-12-21

**Authors:** Anne Cappola

**Affiliations:** University of Pennsylvania School of Medicine, Philadelphia, Pennsylvania, United States

## Abstract

Testosterone and estrogen have anabolic effects, and alterations could affect recovery from hip fracture. We hypothesized that differences in sex hormones during the year after a hip fracture would be associated with bone mineral density (BMD) and physical function. This analysis included 149 women aged 65 years or older enrolled in the Baltimore Hip Studies 7th cohort. Testosterone and estradiol by LC/MS/MS and sex hormone binding globulin were assayed at four time points post hip fracture (within 15 days of admission, and at 2, 6, and 12 months) on fasting morning samples. Longitudinal regression models accounting for clustering by subject were constructed to examine the relationship between each hormone and BMD and short performance physical battery (SPPB) score over time. Mean age was 81 years, BMI was 25.6 kg/m2, and 92% were white. Testosterone levels were low in women at all time points (median 10-12 ng/dL), as were estradiol levels (median 3-5 pg/mL). In adjusted models, increases in total and free estradiol during recovery were associated with improvement in BMD, whereas total testosterone, but not free testosterone, was associated with improvement in SPPB. These analyses support physiologically relevant relationships between higher estradiol levels and improvement in BMD and higher testosterone levels and improvement in physical function during the year after hip fracture, even at the low levels of sex hormones found in post-menopausal women. These findings suggest that differences in reproductive aging that affect sex hormone production may have clinical effects on recovery from a sentinel event, long after menopause.

